# Dental malocclusion at the navel of the Xingu river

**DOI:** 10.1590/2176-9451.19.3.015-016.edt

**Published:** 2014

**Authors:** David Normando

**Affiliations:** editor-in-chief (davidnormando@hotmail.com).

"And whatever is now disclosed to the people, Will surprise everyone not for being exotic, but for having remained concealed Until it was perceived as obvious."

Caetano Veloso, song: "An indian"

The feeling of coexisting with and treating a highly prevalent disease of which origin is unknown is unsettling. It reminds us of when heavy drugs were used to treat cancer of which association with Genetics was still unknown - in fact, nothing was known about the fact that the origin of cancer was right there, in the nucleus of the cell, in the DNA. And this logically resulted in a waste of time and lives. However, when the time came for oncologists and geneticists - cloistered in their laboratories, traveling into the nucleus of the cell - to meet, science made a gold-medal jump and Oncogenetics, the new science, sat on the front chairs of Oncology conferences.

Although millions of people undergo orthodontic treatment, the causes of malocclusion have not yet been clearly determined. That certainly is a paradox for a contemporary world that strives to offer evidence of facts. What is even worse is to know that discussions on malocclusion, held in orthodontic events, raise significantly less interest than presentations about new material and new treatment techniques. Would we be walking towards the same abyss Medicine, a science that does not admit delays, has fallen into?

It all began with Begg^2 ^who, in 1954, studied the skulls of Australian aborigines. He established that malocclusion is "a disease of modern civilization" caused by tooth wear. Understanding Begg's findings means perceiving the brilliant history that influenced the origins of Orthodontics and provided the road that enabled extraction of permanent teeth for space opening in the dental arch so as to counterbalance the absence of interproximal wear. Nevertheless, Begg's concepts went beyond technicality, which bothers a lot of people, as Omar Gabriel would say. A macro analysis reveals the remarkable influence of his theory over the hypothesis that malocclusion, especially dental crowding, is mainly of environmental etiology.

Begg's theory is still controversial and has been discussed under four models of investigation: 1) experiments with animal models; 2) analysis of ancestors' skulls gleaned by means of excavations, and epidemiological studies conducted with primitive/isolated populations; 4) studies conducted with human twins. Overall, the findings have given support to the hypothesis that malocclusion is basically of environmental etiology.

Nevertheless, the issue is not as simple as it may seem and, for this reason, must be brought to light. One should assume that a given malocclusion probably has different etiological factors in comparison to other occlusal alterations. The fact that the obvious may be concealed intrigues a whole generation of uneasy orthodontists who believe that if we repeat the same mistakes made by Oncology - restrain the micro look taken at the nucleus of the cell and the DNA - we may pay a high price for a torpid progress.

Following Begg's line of reasoning, I decided to provoke the monolith of orthodontic architecture by trying to reproduce his finding under a micro look: the DNA. I chose to study the indigenous people from the Brazilian Amazon^3,4^ following the pattern established by Begg. I sailed down the profuse Xingu river, carrying Genetics in my saddlebag.

Initially, it is worth noting that it is common for the Amerindian (American indigenous) to establish new settlements, whether by means of fission (division) or fusion (union). Thus, it is easy to understand that miscegenation contributes to increasing genetic variation among indigenous people, which counterbalances with low intratribal variation.^5^ Therefore, unlike what it is commonly believed, indigenous groups are genetically different from each other, even though the individuals comprising the same village are similar in appearance.

Would that be the perfect opportunity for me to answer my questions regarding the theme? I put on my indigenist boots and my researcher saddlebag and, just like Begg who is now disguised in the brazilianity of a Villas-Bôas, I steered my horse towards the Xingu river where I remained between 2009 and 2010. The area has several settlements consisted of indigenous people of nine different ethnic groups. It is, therefore, one of the most ethnically diverse human community in the world.

During our first trip, we established contact with two Arara villages. Thus, two genetically different settlements.

An anthropological study about the Arara people^6^ revealed that the individuals comprising the Arara of Iriri village come from the same family who descend from a couple who, according to historical reports, was expelled from the Arara of Laranjal village. The separation process supposedly occurred between 1925 and 1926 when the indigenous people lived isolated.

The expelled couple had seven children and the initial expansion of the Arara of Iriri village occurred through the mating of closely related people characterized as incestuous (brother-sister, father-daughter, mother-son). Afterwards, it was established as a result of marriage between close relatives (aunt-nephew, uncle-niece, first cousins).^5^ Inbreeding, however, was rare in the ancestral tribe.

A genetic study^5^ comparing Arara villages revealed the presence of one single haplotype DNAY and mitochondrial DNA, which corroborates the historical reports on the descendant group. The study also revealed a drastic case of linear fission (involving relatives) within an ancestral tribe. Furthermore, it revealed that the genetic distance and the molecular variance between the two Arara villages were so great that despite having the same origin, their populations significantly differ in terms of genotype.

These indigenous people keep the same traditional eating habits, which was proved by similar tooth wear (a direct evidence of what an individual ate in the past) found between different villages. Therefore, both populations have the same origin and live in similar environments, but they are significantly different in terms of genetics. That was an open door for answering my initial question, but now, with a genetic support.

In the study design, we obtained an epidemiological assessment of malocclusion prevalence, which revealed marked differences in the dentofacial characteristics of the tribes. Malocclusion was twice as prevalent in the descendent village (Arara of Iriri). Most individuals comprising the original villages had a normal occlusal relationship, whereas those comprising the descendent group often had Class III malocclusion. The condition affected one third of the population. Another spasmodic result was the absence of dental crowding in the descendent village, while the original group had one fourth of its population affected by this type of malocclusion. These results mitigate the influence of tooth wear on dentofacial development and highlight the predominance of heredity in the etiology of abnormal variation in dental occlusion .We went on the opposite direction of the river rapids imported by Begg's findings.^2^

After my journey on the land of the Arara, I decided to steer my canoe even further. That was when we decided to visit the Assurini people in the Xingu river, and two Xicrin-Kaiapó villages in the Bacajá river. The results yielded by our investigation confirmed the marked difference in the occlusal pattern of these groups. We reached the same conclusion. No great news for Dr. Cléber Bidegain Pereira, a Brazilian orthodontist from Uruguaiana who visited the Yanomamis in the 70s. The Yanomamis lived in complete isolation from civilization. Notwithstanding this fact, the results yielded by Dr. Pereira revealed that 71% of the sample had some feature of malocclusion, similarly to what is found for the Brazilian urban population. Crowding was observed in 48% of the sample, even though the indigenous population kept their traditional eating habits. Adults had completely worn cusps. Our true Villas-Bôas of Orthodontics concluded that malocclusion is genetically determined and follows the morphological human evolution. Thus, it does not rely on an individual's masticatory activity. We reached the same village half a century later.

Every science must accept the potential errors arising from its conclusions: that is a fact. Science must also establish a reasonable amount of self-contemplation, often examining its exposed navel - a common habit among indigenous people. There is a chance that we are mistaken. But black swans are real in the scientific community as well as in non-fiction works, as it is the case of the movie directed by Cao Hamburger - a moviemaker generated by scientists. Xingu: a confluence of water, people, science and conscience.
